# The impact of COVID-19 lockdown announcements on mental health: quasi-natural experiment in Lombardy, Italy

**DOI:** 10.1093/eurpub/ckac035

**Published:** 2022-04-12

**Authors:** Yuxi Wang, Alessandra Lugo, Andrea Amerio, Luca Cavalieri d’Oro, Licia Iacoviello, Anna Odone, Alberto Zucchi, Silvano Gallus, David Stuckler, the Lost in Lombardia Project Investigators, Silvano Gallus, Cristina Bosetti, Carlotta Micaela Jarach, Alessandra Lugo, Chiara Stival, Gianluca Serafini, Andrea Amerio, Mario Amore, David Stuckler, Roberto De Sena, Simone Ghislandi, Yuxi Wang, Licia Iacoviello, Marialaura Bonaccio, Francesco Gianfagna, Anwal Ghulam, Anna Odone, Carlo Signorelli, Paola Bertuccio, Giansanto Mosconi, Giacomo Pietro Vigezzi, Luca Cavalieri d'Oro, Magda Rognoni, Marco Scala, Alberto Zucchi, Roberta Ciampichini

**Affiliations:** Department of Social and Political Science, Dondena Centre for Research on Social Dynamics and Public Policy, Bocconi University, Milan, Italy; Department of Environmental Health Sciences, Istituto di Ricerche Farmacologiche Mario Negri IRCCS, Milan, Italy; Department of Neuroscience, Rehabilitation, Ophthalmology, Genetics, Maternal and Child Health, Section of Psychiatry, University of Genoa, Italy, Genoa; IRCCS Ospedale Policlinico San Martino, Genoa, Italy; Epidemiology Unit, Agenzia per la Tutela della Salute—ATS—della Brianza (Local Public Health Authority), Monza, Italy; Department of Medicine and Surgery, Research Center in Epidemiology and Preventive Medicine (EPIMED), University of Insubria, Varese, Italy; Department of Epidemiology and Prevention, IRCCS NEUROMED, Pozzilli, Italy; Department of Public Health, Experimental and Forensic Medicine, University of Pavia, Pavia, Italy; Epidemiology Unit, Agenzia per la Tutela della Salute—ATS—di Bergamo (Local Public Health Authority), Bergamo, Italy; Department of Environmental Health Sciences, Istituto di Ricerche Farmacologiche Mario Negri IRCCS, Milan, Italy; Department of Social and Political Science, Dondena Centre for Research on Social Dynamics and Public Policy, Bocconi University, Milan, Italy

## Abstract

**Background:**

Evidence showed that mental health problems have risen markedly during COVID-19. It is unclear if part of the mental sufferings relates to the climate of uncertainty and confusion originated from rough communication by health officials and politicians. Here, we test the impact of unanticipated policy announcements of lockdown policies on mental health of the older population.

**Methods:**

We used a representative telephone-based survey of 4400 people aged 65 years or older in Italy’s Lombardy region to compare information on self-reported symptoms of anxiety, depression and poor-quality sleep of subjects interviewed on the days of the policy announcement with that of subjects interviewed on other days. We used regression models adjusting for potential socio-demographic confounders as well study design with inverse probability weighting.

**Results:**

On days when policymakers announced to extend the lockdown, mental health deteriorated on average by 5.5 percentage points [95% confidence interval (CI): 1.1–9.8] for self-reported anxiety symptoms and 5.1 percentage points (95% CI: 2.7–7.4) for self-reported depressive symptoms. The effect of the announcement to shorten the lockdown is more moderate but statistically significant. These associations were short term in duration; after just 1 day, self-reported mental health and sleep quality return to levels better than pre-announcement until a new policy change.

**Conclusions:**

Our research shows that lockdown policy announcements are associated with short-term worsening in mental distress, highlighting the importance of appropriate communication strategies and political determinations in crisis times.

## Introduction

There are widespread concerns that the COVID pandemic and associated policy lockdowns could cause a deterioration in mental health.^1–4^ UK and Italian surveys from earliest stages of the COVID outbreak from March to May 2020 reveal large increases in the numbers of people experiencing symptoms of depression and anxiety.^1^^,^^5^ More recent European and North American studies report that these increases are not just among those with a history of depression but also among persons who had no sign of pre-existing mental health problems.^2^^,^^6–9^

It is unclear why mental health is worsening. One leading hypothesis is that generalized fear of the virus is triggering increased anxiety. Another is that government-imposed restrictions on people’s mobility and personal freedom constrain their autonomy and as a result increase depression risk. Often these two explanations are invoked interchangeably, but recent research has begun to disentangle them. Italian researchers found that mental health worsened not with the onset of COVID but during the first wave of lockdown measures in May 2020.^7^^,^^10^ Another US study identified how declines in mobility, arising from lockdown measures, were positively correlated with mental distress.^11^

Yet there is a third, as to our knowledge yet untested, possibility: could government announcements of impending lockdowns provoke anxiety and depression? It is possible that sudden and frequent changes in lockdown policies could cause people to feel frustrated, uncertain about their future, and lacking control over their lives, all of which are well-known psychological risk factors for depression and anxiety.^12^^,^^13^ Indeed, one well-established finding from social epidemiology is that the fear of an adverse event, such as a job loss, can be worse for mental health than the event itself, such as unemployment.^14^

Here, we aim to plug this gap by testing the hypothesis that unanticipated lockdown announcements worsen mental health. Our analysis applies a quasi-natural experimental framework, drawing on the Italian government’s lockdown announcements in the Lombardy region, which was one of the earliest and hardest hit centres of the COVID-19 pandemic. Supplementary appendix box S1 provides further detail about the Italian context. In brief, in the observation period of our study, there were two major announcements by the central government regarding policy changes in lockdown measures. The announcements are unanticipated because the residents are only told on the same day that a press conference will take place, and there is no prior communication on the possible policy changes. Specifically, we test our hypothesis using cross-sectional data covering pre- and post-periods of lockdown announcements for the older adults’ population, who are widely believed to be among the most vulnerable.

## Methods

### Sources of data

We designed a telephone-based survey, which was conducted by Doxa, the Italian branch of the worldwide independent network/Gallup International Association and coordinated by Mario Negri Institute within the project LOckdown and lifeSTyles in Lombardia (Lost in Lombardia). This created a unique dataset containing a representative sample of 4400 subjects aged 65 years or more from Lombardy region of Italy who were interviewed between 17 and 30 November 2020.^15^

Participants of the survey were randomly recruited and are representative of Lombardy region. Multistage sampling was applied to ensure the representativeness of the older population (65 years or older). In brief, from the entire household population in Lombardy, we randomly selected 30 000 families as the primary sampling units according to the town size quota in the region. Then, among the 30 000 families, the subjects to be interviewed are selected according to the gender, age and education quotas as proportion to the total population. Finally, the data processing that involves generating statistical weights for each subject is performed. The overall response rate is around 42% among the sample of representative households and is evenly distributed across the interview dates. The questionnaire captured participants’ main socio-demographic information, including their age, sex and marital status, socioeconomic characteristics such as residence province, household size, level of education, self-reported income, as well as whether he/she had contracted COVID-19 or had been diagnosed with other chronic conditions.

### Outcome measure

We assessed respondents’ probable caseness for depression, anxiety and sleep disorders with a series of scales validated for use in non-clinical settings: *anxiety symptoms* using the 2-item Generalized Anxiety Disorder scale (GAD-2)^16^; *depressive symptoms* using the 2-item Patient Health Questionnaire (PHQ-2)^17^; *poor sleep quality* using one key question from the Pittsburgh Sleep Quality Index (PSQI).^18^ Following standard approaches, we considered a person to exhibit probable caseness for anxiety (GAD-2) or depression (PHQ-2) scores were ≥3.^19^ Poor sleep quality was defined as those rated their overall quality of sleep as ‘quite bad’ or ‘very bad’, corresponding to scores equal to or higher than 3. Supplementary appendix box S2 shows all verbatim questions employed.

### Quasi-natural experiment design

To better ascertain causality, we employed a quasi-natural experiment design taking advantage of date cut points in lockdown announcements. Supplementary appendix figure SA1 shows the specific dates of the survey interviews and the major lockdown announcements. On 20 November, one government ordinance extended strict lockdown for five more days. Then again on 28 November, the Italian government changed its position and shortened the duration of lockdown. Both changes were unanticipated and are likely to have provoked a sense of confusion and uncertainty.

Our research design takes advantage of these unexpected policy changes to quantify the association between these announcements and symptoms of anxiety, depression and poor sleep quality. Since a random sampling procedure was performed, we simulate a randomized controlled trial by creating a random assignment pattern of some people being exposed to the announcement and others who were not, based on survey dates. Effectively this creates a ‘treatment group’ of those surveyed on announcement dates and a ‘control group’ of all others surveyed on non-announcement dates. Because the two announcements conveyed different lockdown messages, we divide our sample population to analyze the treatment effects separately.

In addition, we evaluate the potential ‘rebound’ from the announcement on mental health. In doing so, we compared the average outcomes of those surveyed post-announcement (treatment group) to those surveyed pre-announcement (control group).

### Balance test

To verify that this resulted in a randomized pattern distributing respondents into these treatment and control groups, just like for randomized-controlled trials we performed a ‘balance test’.

The number of observations across interview dates can be found in the Supplementary appendix table SA1, while Supplementary appendix tables SA2–SA4 show the balance tests of respondents’ characteristics across treatment and control groups. For the sample around first announcement (Supplementary appendix tables SA2 and SA4), we observe no systematic difference for all observable characteristics between our treatment and control group. Due to sampling limitations, from 28 November on there was insufficient number of older persons in the sample cell of below 75 years old. To ensure comparability across all sampling days for the second announcement, we exclude this age group (65–74) for both control and treatment group to ensure an unbiased estimation of the treatment effect. After this exclusion, we see that the sample around the second announcement is still unbalanced in terms of respondents’ gender, education, civil status and COVID history (Supplementary appendix table SA3). We, therefore, include these demographic variables in the analysis as covariates for the treatment probability as discussed in the following section.

### Statistical model

We estimated the association of lockdown policy announcement with symptoms of anxiety, depression and poor sleep quality using the following statistical model:

$${\mathrm{Mental}\;\mathrm{Health}}_{i}=\beta_{0}+\beta_{1}a\mathrm{nnouncement}\;\mathrm{date}+\gamma_{i}+\epsilon_{i},$$

whereby ${\mathrm{Mental}\;\mathrm{Health}}_{i}$ is a vector of above-mentioned mental health outcome variables, announcement date is a dummy that equals to 1 if the respondent is interviewed on the announcement days and 0 otherwise, $\beta_{1}$ is, therefore, the treatment effect of announcement, $\gamma_{i}$ is a vector of controls and $\epsilon_{i}$ is the error term. Robust standard errors are clustered at the provincial level. The control variables included age category, gender, residence province, education level, civil status, income level, having past COVID-19 infection, living in a single household and having chronic illness.

One potential problem that arises in the analysis is sampling bias in the interview responses. In theory, our respondents should be randomly selected on all interview days and therefore should not create bias in the characteristics of the respondent. However, the respondents’ characteristics may confound the probability of responding to the telephone interview on the announcement days. In this case, the mental health outcome and the announcement treatment are not necessarily independent, and estimations can be biased.

To deal with this issue, we estimate the average treatment effect on mental health accounting for the probability of responding to the interview. In doing so, we use the inverse-probability weighting (IPW) estimator to account for the potential bias from the sampling process.^20^ We combine the covariate information into the estimated treatment probability by reweighting the respondents according to all observed characteristics, including gender, age, education, civil status, income level and health status. This reweighting will allow the same distributions of each covariate between treatment and control groups and, in so doing, adjusting for their potentially confounding effects. Supplementary appendix box S2 further describes the potential-outcome framework and the IPW approach. In analyzing the immediate effect of announcement days, the treatment groups include those interviewed on announcement days (20 and 28 November) and the control groups include those interviewed on all other days before and after the announcements. For the rebound effect, we consider the treatment group as those interviewed post-announcement and the control group those interviewed pre-announcement. Because we do not have sufficient data points after the second announcement, we analyze the rebound effect only for the first announcement.

Because the announcement days occurred on Friday (20 November) and Saturday (28 November), one might argue that the cyclical pattern across the days of the week can confound the result. In fact, existing literature shows that on weekdays, individuals tend to exhibit worse subjective mental health than on weekends.^21^^,^^22^ If we find worsened mental health on the announcement days, which occurred on weekends, the results can further strengthen our argument because the identified effect will be the lower bound. As a robustness check, we include the fixed effects of each interview day to account for the potential effects of ‘day of the week’. We also employ a non-linear probit model that includes each interview day as a dummy variable for the mental health outcomes, adjusting for all the covariates. We use the day with the lowest mean as the reference day. In this way, we can obtain the marginal effect of each interview day on our outcome variables to see if our main results still hold.

### Ethics approval

The protocol of the study was approved by the ethics committee (EC) of the coordinating group (EC of Fondazione IRCCS Istituto Neurologico Carlo Besta, File number 76, October 2020). All the participants provided their consent to participate in the study.

## Results

### Descriptive statistics

**Figure 1:**
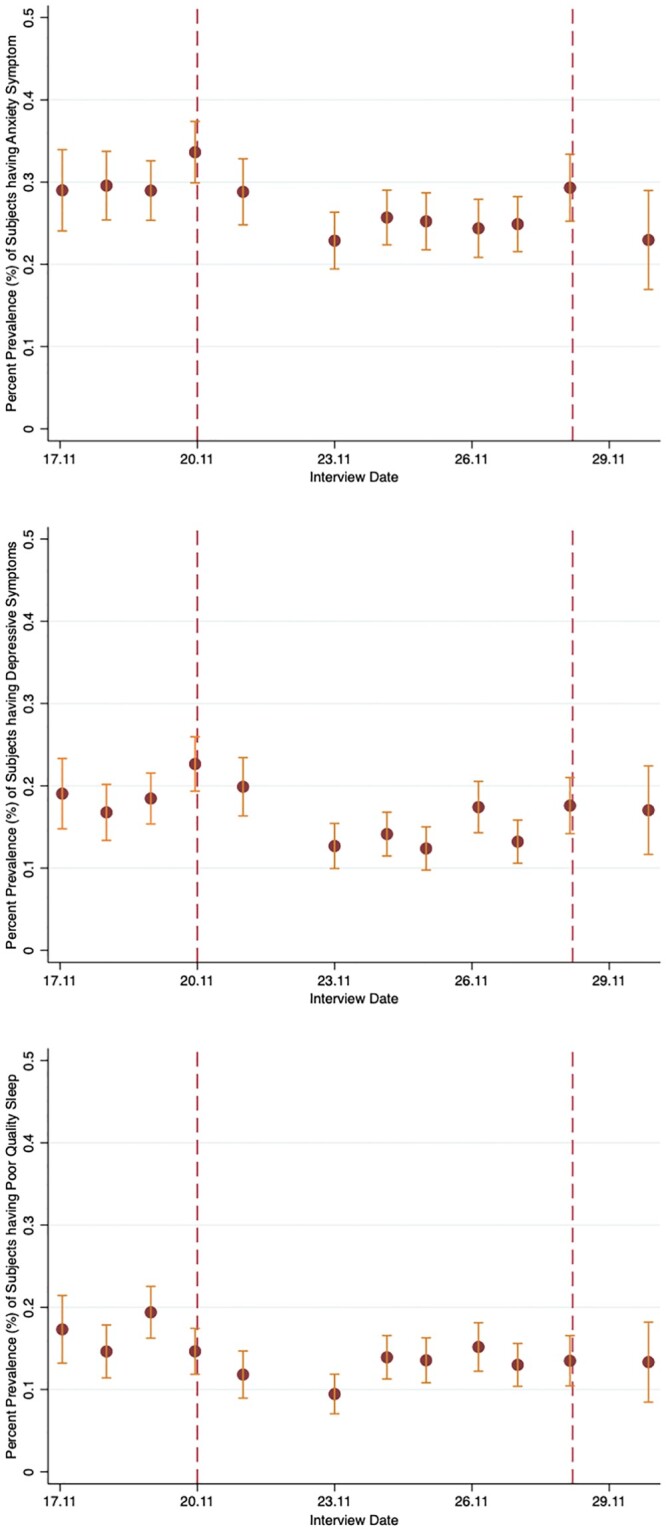
Trends in levels of anxiety symptoms, depressive symptoms and poor-quality sleep, 17–30 November 2020. *Notes:* This figure reports the percentage prevalence (%) of subjects having anxiety symptoms, depressive symptoms and poor-quality sleep and their 95% CIs conditioning on the interview date. The vertical-dotted reference lines indicate the announcement days

Figure 1 shows the trends in anxiety symptoms, depressive symptoms and sleep quality from 17 November to 30 November. We see that the means of the mental health variables are considerably higher on the two announcement days (20 and 28 November) compared with other days, indicating that more people exhibit anxiety and depressive symptoms on those days. The trend is not as obvious for quality of sleep. There is also a visible rebound effect as mental health symptoms tend to improve in the period following the announcements.

### Impact of the lockdown policy announcements: immediate and rebound effects

First, we looked at the immediate effect of the policy announcement on mental health. Table 1 shows the unadjusted, risk-adjusted and IPW estimators of the announcement treatment effect. We observe that more people exhibit anxiety and depressive symptoms across all three models on the announcement days. After reweighting (the IPW model), we see that the rate of respondents exhibiting anxiety symptoms is about 5.5 percentage points [95% confidence interval (CI): 1.1–9.8] higher for the first announcement and about 5.0 percentage points (95% CI: 0.6–9.3) higher for the second announcement day than other days. For depressive symptoms, the effect size is about 5.1 percentage points (95% CI: 2.7–7.4) for the first announcement and about 3.3 percentage points (95% CI: 0.5–6.1) for the second announcement. There was no significant effect on the quality of sleep. The full models of table 1 can be found in the Supplementary appendix tables SA5 and SA6.

**Table 1:** Association of announcement days with anxiety symptoms, depressive symptoms and poor-quality sleep

		(1)	(2)	(3)
Models		Anxiety symptoms	Depressive symptoms	Poor-quality sleep
First announcement	Unadjusted	0.064^**^	0.061^***^	0.003
		(0.013 to 0.115)	(0.025 to 0.098)	(−0.049 to 0.056)
	Adjusted for confounding factors	0.053^**^	0.052^***^	0.007
		(0.008 to 0.097)	(0.023 to 0.082)	(−0.040 to 0.055)
	IPW	0.055^**^	0.051^***^	0.005
		(0.011 to 0.098)	(0.027 to 0.074)	(−0.040 to 0.049)
	Number of respondents	2640	2640	2640
Second announcement	Unadjusted	0.030	0.021	−0.020
		(−0.023 to 0.083)	(−0.008 to 0.050)	(−0.048 to 0.008)
	Adjusted for confounding factors	0.055^**^	0.033^**^	−0.014
		(0.008 to 0.102)	(0.003 to 0.063)	(−0.042 to 0.015)
	IPW	0.050^**^	0.033^**^	−0.021^*^
		(0.006 to 0.093)	(0.005 to 0.061)	(−0.043 to 0.001)
	Number of respondents	1207	1207	1207

Robust standard errors clustered at provincial level; standard errors in parentheses. Outcome measurement units: having anxiety symptom, having depressive symptom and having poor-quality sleep.

*** *P* < 0.01,

** *P* < 0.05,

* *P* < 0.1.

In understanding the rebound effect for the first announcement, we estimate the treatment effect of post-announcement on the same set of mental health variables. As seen in table 2, on average, people interviewed after the first announcement have 5.5 percentage points (95% CI: −8.5 to −2.4) lower rate of anxiety than those interviewer before the first announcement. The effect size for depressive symptoms is about 4.6 percentage points (95% CI: −6.9 to −2.2) lower for the treatment group. Moreover, the rate of people having poor-quality sleep is lowered by about 4.7 percentage points (95% CI: −6.8 to −2.6) after the announcement. The full models of table 2 can be found in Supplementary appendix table SA7.

**Table 2:** Association of post-announcement with anxiety symptoms, depressive symptoms and poor-quality sleep

		(1)	(2)	(3)
	Models	Anxiety symptom	Depressive symptom	Poor-quality sleep
Post-Announcement	Unadjusted	−0.049^**^	−0.042^**^	−0.047^***^
		(−0.085 to −0.013)	(−0.072 to −0.011)	(−0.070 to −0.023)
	Adjusted for confounding factors	−0.057^***^	−0.048^***^	−0.047^***^
		(−0.089 to −0.026)	(−0.074 to −0.022)	(−0.071 to −0.022)
	IPW	−0.055^***^	−0.046^***^	−0.047^***^
		(−0.085 to −0.024)	(−0.069 to −0.022)	(−0.068 to −0.026)
	Number of respondents	2640	2640	2640

Robust standard errors clustered at provincial level; standard errors in parentheses. Outcome measurement units: having anxiety symptom, having depressive symptom and having poor-quality sleep.

*** *P* < 0.01,

** *P* < 0.05,

* *P* < 0.1.

### Robustness checks

**Figure 2:**
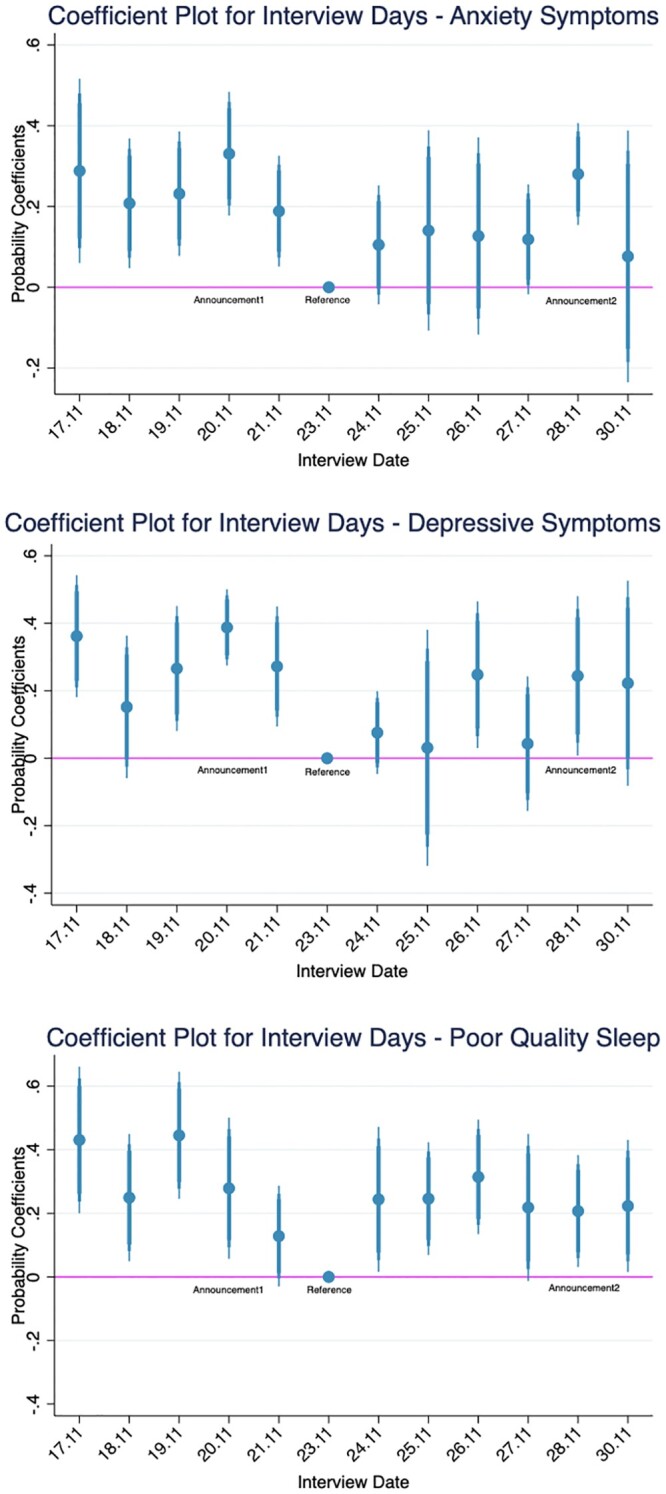
Coefficient plots for interview days from probit model after risk adjustment. *Notes:* This figure reports the coefficient plots with 95% confidence interview from the probit model from Supplementary appendix table SA9. The horizontal line represents the reference level of the lowest mean of the outcome variable across all interview dates

First, in analyzing the immediate effect of the announcement, we include a set of interview day fixed effects to account for the potential ‘day of the week’ effect as discussed. As seen in Supplementary appendix table SA8, after the inclusion of interview days, our results using the IPW remain significant and the magnitude did not change drastically. In our second robustness check, we created a set of dummy variables for all interview days to understand the marginal effects of each observable day on our outcome variables, controlling for observable respondent characteristics. As seen in Supplementary appendix table SA9, on 20 November and 28 November, the marginal effects on anxiety and depressive symptoms are significant and positive compared with the reference date. In fact, the effects are the strongest on the announcement dates for anxiety symptoms. Although the marginal effect for the second announcement date is not as large for depressive symptoms, the effect is nonetheless significant. The rebound effect can also be observed across the three variables, as there is a gradual drop in the marginal effect after the first announcement. As seen in the coefficient plots of figure 2, just 1 day after the announcement, the rate of anxiety and depressive symptoms immediately drops to a level lower than pre-announcement days, and the coefficients remain low until the second announcement. Moreover, those who have income well below the Italian average, have contracted COVID-19, or have chronic diseases exhibit worse mental health (Supplementary appendix table SA9). The coefficients for other covariates can be found in Supplementary appendix table SA10.

## Discussion

Our study looks at how unexpected lockdown policy changes are associated with mental health outcomes of the older population. We show that on the announcement day to extend the lockdown, symptoms of anxiety are about 5.5 percentage points (95% CI: 1.1–9.8) higher than other days, and symptoms of depression are 5.1 percentage points (95% CI: 2.7–7.4) higher than days before and after. The association between the second announcement to shorten lockdown and mental health outcomes are around 5.0 percentage points (95% CI: 0.6–9.3) higher for symptoms of anxiety and 3.3 percentage points (95% CI: 0.5–6.2) for symptoms of depression. Moreover, after the first announcement, anxiety and depressive symptoms are reduced by around 5.5 percentage points (95% CI: −8.5 to −2.4) and 4.6 percentage points (95% CI: −6.9 to −2.2) compared with the level before and on the announcement day. The average number of people having poor-quality sleep is lowered by about 4.7 percentage points (95% CI: −6.8 to −2.6) after the announcement. This improvement in mental health symptoms occurs as early as 1 day after the announcement, and the overall level of mental health is higher on average than pre-announcement. Because participants were already in lockdown since the beginning of the interview period, we believe this level shift of mental health represents an overall improvement of anxiety and depression symptoms once knowing when the lockdown will end. Our results imply that uncertainty and the constant change in lockdown policies can be a crucial driver for temporary mental distress, and once certainty is established, the symptoms improve.

Our study has several limitations. First, we do not have a very long observation time frame that allows us to run a time series analysis over the entire lockdown period for an even more robust analysis. This will be hard to achieve given the short-lived lockdown policies and varying degrees of restrictions at different periods. We offer, however, cleaner analysis of the effect of policy changes in a single period. By relying on the region-wide survey and naturally occurring events, the external validity of our findings is higher than in randomized control trials. Second, since our respondents answered the survey in a non-clinical setting via telephone, our mental health outcome variables are inevitably subjective measures. Moreover, the exact questionnaire refers to the frequency of mental health symptoms over the course of 2 weeks. Nonetheless, what we captured in the analysis is the average effect of the self-reported mental health outcome, controlling for all the individual-level confounding factors. In any case, if we consider the outcome variables as the average value over the 2 weeks, our results will represent an underestimation of the actual effect of the announcement on mental health. Third, we do not observe the exact time when the interview is conducted, which means that we do not know whether the respondent is interviewed before or after the announcement press conference. Nonetheless, respondents interviewed during the day should be aware that a press conference is to take place at 8 pm given it is the headline of most newspapers, but it is likely that the interview is conducted before the evening announcement. Therefore, we can reasonably assume that the effect we observe on the press conference day is the announcement effect and not a post-announcement effect. Moreover, we can confidently assume that all respondents are exposed to the official announcement due to the strict lockdown situation in Lombardy. Finally, we cannot effectively differentiate whether the observed effect is due to the announcement *per se* or to what the new policy entails. What we observe is a combined effect of unexpected policy changes as well as the policy itself. Nevertheless, because we detected significant effects not only from a negative (extending lockdown) announcement but also a positive (lifting lockdown) announcement, we can presume that the announcement *per se* indeed played a role.

The mental health effect from lockdown policy changes, although generally short-lived, can translate to more serious social issues if there is not enough political determination in implementing and lifting restrictive measures. Precisely because of the collective trauma that individuals live through, uncertainty and frequent changes can add a further layer of stress and confusion for the general population. In addition, wavering lockdown policies and unclear communications may potentially lead people to break rules that they do not fully understand or disengage from trying to keep abreast of restriction, which could well lead to lower compliance in the long term. Further studies on the lockdown policies should investigate how the frequency of lockdown policy changes can contribute to longer-term mental health issues for the vulnerable population.

## Supplementary data

Supplementary data are available at *EURPUB* online.

## Supplementary Material

ckac035_Supplementary_Data

## References

[1] Abbott A. COVID’s mental-health toll: how scientists are tracking a surge in depression. Nature 2021;590:194–5. 33536600 10.1038/d41586-021-00175-z

[2] Adams-Prassl A , Boneva T, Golin M, Rauh C. The Impact of the Coronavirus Lockdown on Mental Health: Evidence from the US. Working Papers. Human Capital and Economic Opportunity Working Group; 2020 May (Working Papers). Report No. 2020–030. Available at: https://ideas.repec.org/p/hka/wpaper/2020-030.html (25 March 2021, date last accessed).

[3] Arendt F , Markiewitz A, Mestas M, Scherr S. COVID-19 pandemic, government responses, and public mental health: investigating consequences through crisis hotline calls in two countries. Soc Sci Med 2020;265:113532. 33223385 10.1016/j.socscimed.2020.113532

[4] Moreno C , Wykes T, Galderisi S, et al How mental health care should change as a consequence of the COVID-19 pandemic. Lancet Psychiatry 2020;7:813–24. 32682460 10.1016/S2215-0366(20)30307-2 PMC7365642

[5] Chandola T , Kumari M, Booker CL, Benzeval MJ. The mental health impact of COVID-19 and pandemic related stressors among adults in the UK. medRxiv 2020. doi:10.1101/2020.07.05.20146738 PMC7783135 33280639

[6] Ahrens KF , Neumann RJ, Kollmann B, et al Differential impact of COVID‐related lockdown on mental health in Germany. World Psychiatry 2021;20:140–1. 33432755 10.1002/wps.20830 PMC7801843

[7] Amerio A , Lugo A, Stival C, et al COVID-19 lockdown impact on mental health in a large representative sample of Italian adults. J Affect Disord 2021;292:398–404. 34139414 10.1016/j.jad.2021.05.117 PMC8777065

[8] Kochhar AS , Bhasin R, Kochhar GK, et al Lockdown of 1.3 billion people in India during Covid-19 pandemic: a survey of its impact on mental health. Asian J Psychiatry 2020;54:102213. 10.1016/j.ajp.2020.102213 PMC7301781 32599544

[9] Panchal N , Kamal R. The Implications of COVID-19 for Mental Health and Substance Use. KFF. 2021. Available at: https://www.kff.org/coronavirus-covid-19/issue-brief/the-implications-of-covid-19-for-mental-health-and-substance-use/ (1 June 2021, date last accessed).

[10] Rossi R , Socci V, Talevi D, et al COVID-19 Pandemic and Lockdown Measures Impact on Mental Health Among the General Population in Italy. Front Psychiatry. 2020;11. Available at: https://www.frontiersin.org/articles/10.3389/fpsyt.2020.00790/full?&;utm_source=Email_to_authors_&;utm_medium=Email&;utm_content=T1_11.5e1_author&;utm_campaign=Email_publication&;field=&;journalName=Frontiers_in_Psychiatry&;id=550552 (25 March 2021, date last accessed). 32848952 10.3389/fpsyt.2020.00790 PMC7426501

[11] Devaraj S , Patel PC. Change in psychological distress in response to changes in reduced mobility during the early 2020 COVID-19 pandemic: evidence of modest effects from the U.S. Soc Sci Med 2021 Feb 1;270:113615. 33352476 10.1016/j.socscimed.2020.113615 PMC9757908

[12] Goldsmith AH , Veum JR, William D. The impact of labor force history on self-esteem and its component parts, anxiety, alienation and depression. J Econ Psychol 1996;17:183–220.

[13] Molarius A , Berglund K, Eriksson C, et al Mental health symptoms in relation to socio-economic conditions and lifestyle factors—a population-based study in Sweden. BMC Public Health 2009;9:302. 19695085 10.1186/1471-2458-9-302 PMC2736164

[14] Reichert AR , Tauchmann H. The Causal Impact of Fear of Unemployment on Psychological Health. Rochester, NY: Social Science Research Network; 2011. Report No. ID 1880938. Available at: https://papers.ssrn.com/abstract=1880938 (2 September 2021, date last accessed).

[15] Odone A , Lugo A, Amerio A, et al COVID-19 lockdown impact on lifestyle habits of Italian adults. Acta Bio Medica Atenei Parm 2020;91:87–9. 10.23750/abm.v91i9-S.10122 PMC8023096 32701921

[16] Kroenke K , Spitzer RL, Williams JBW, et al Anxiety disorders in primary care: prevalence, impairment, comorbidity, and detection. Ann Intern Med 2007;146:317–25. 17339617 10.7326/0003-4819-146-5-200703060-00004

[17] Kroenke K , Spitzer RL, Williams JBW. The Patient Health Questionnaire-2: validity of a two-item depression screener. Med Care 2003;41:1284–92. 14583691 10.1097/01.MLR.0000093487.78664.3C

[18] Buysse DJ , Reynolds CF, Monk TH, et al The Pittsburgh Sleep Quality Index: a new instrument for psychiatric practice and research. Psychiatry Res 1989;28:193–213. 2748771 10.1016/0165-1781(89)90047-4

[19] Staples LG , Dear BF, Gandy M, et al Psychometric properties and clinical utility of brief measures of depression, anxiety, and general distress: the PHQ-2, GAD-2, and K-6. Gen Hosp Psychiatry 2019;56:13–8. 30508772 10.1016/j.genhosppsych.2018.11.003

[20] Wooldridge JM. Inverse probability weighted M-estimators for sample selection, attrition, and stratification. Port Econ J 2002;1:117–39.

[21] Helliwell JF , Wang S. Weekends and subjective well-being. Soc Indic Res 2014;116:389–407.

[22] Zawadzki MJ , Scott SB, Almeida DM, et al Understanding stress reports in daily life: a coordinated analysis of factors associated with the frequency of reporting stress. J Behav Med 2019;42:545–60. 30600403 10.1007/s10865-018-00008-x PMC6526071

