# Supplementary material for: The impact of COVID-19 lockdown announcements on mental health: quasi-natural experiment in Lombardy, Italy
Source: Eur J Public Health. 2022 Apr 12;32(3):488–93. doi: 10.1093/eurpub/ckac035 (PMC9159307; doi:10.1093/eurpub/ckac035)
Supplement: ckac035_Supplementary_Data [file ckac035_supplementary_data.docx]

**Appendix:**

**Box 1. Lombardy’s lockdown and COVID epidemic**

Lombardy, the most populated and industrialised Italian region, was among the epicentres of the very first wave of coronavirus disease 2019 (COVID-19) in the world. At the beginning of the pandemic, the healthcare system was rapidly swamped by the surge of hospitalisation. Notwithstanding public pressure, Italy became the first western country to announce a countrywide lockdown on the March 9^th^, 2020, with public places and workplaces closed and people forced to stay at home throughout April and May.

The heralded second wave has brought about more moderate yet confusing lockdown measures. As cases continued to rise, in early November, the Italian government introduced a new regulation^[[1]](#footnote-1)^ that divided each Italian region into three colour categories according to their local risk as measured by pooling 21 indicators that are not publicly known. On November 6^th^, 2020, the Lombardy region was assigned the colour red (red zone), which is the most restrictive with all movement prohibited except for work, health or other urgent reasons. Although the lockdown was enacted to last until November 28^th^, on November 20^th^, an announcement signed on the previous day^[[2]](#footnote-2)^ extended the red zone for Lombardy until December 3^rd^. Yet on November 28^th^, a new announcement signed on the day before^[[3]](#footnote-3)^ stipulated that Lombardy will be entering orange zone (less restrictive than the red zone, indicating a medium level of restrictions) from November 29^th^. These announcements are unanticipated because there is no communication prior to the day of announcement regarding the press conference. We want to exploit this specific ordinance of lengthening the red zone for Lombardy to understand how the mental stability of the elderly population is disturbed by the unexpected change in lockdown policies and the differential effects across levels of vulnerabilities. The details of the timeline are presented in Figure A1.

**Box 2. Interview Questions**

*Symptoms of Anxiety:*

- How often are you bothered by feeling of nervous, anxious and tense?
  - Never (1)
  - Some days (2)
  - Often (3)
  - Almost every day (4)
- How often are you constantly worrying or concerned?
  - Never (1)
  - Some days (2)
  - Often (3)
  - Almost every day (4)

*Symptoms of Depression:*

- How often have you felt little interests or pleasure in doing things?
  - Never (1)
  - Some days (2)
  - Often (3)
  - Almost every day (4)
- How often do you feel depressed or hopeless?
  - Never (1)
  - Some days (2)
  - Often (3)
  - Almost every day (4)

*Quality of Sleep:*

- How do you assess the overall quality of your sleep?
  - Very good (1)
  - Pretty good (2)
  - Pretty bad (3)
  - Very bad (4)

**Box 3. Potential Outcome Framework and Inverse Probability Weighting**

The potential-outcome model (POM) describes the observed mental health outcome variable as ${MH}_{0}$when treatment variable $t=0$(respondent interviewed on the announcement day) and ${MH}_{1}$ when $t=1$ (respondent interviewed on non-announcement day). Algebraically, we have

$MH=(1-t){\cdot MH}_{0}+t\cdot{MH}_{1}$.

The $ATE$ is defined as:

$$\tau_{ATE}=E({MH}_{1}-{MH}_{0})$$

The functional forms for ${MH}_{0}$ and ${MH}_{1}$ are:

$${MH}_{0i}=\boldsymbol{\beta}_{\boldsymbol{0}}\boldsymbol{\gamma}_{\boldsymbol{i}}+\epsilon_{0i}$$

$${MH}_{1i}=\boldsymbol{\beta}_{\boldsymbol{1}}\boldsymbol{\gamma}_{\boldsymbol{i}}+\epsilon_{1i}$$

Where $\beta_{0}$ and $\beta_{1}$ are the coefficients of the covariates, and $\epsilon_{0}$ and $\epsilon_{1}$ are the error terms. And the treatment assignment is

$$t_{i}=\left\{ \begin{aligned} 1 if \gamma\boldsymbol{w}_{\boldsymbol{i}}\boldsymbol{+}\eta_{i}>0 \\ 0 otherwise \end{aligned} \right.$$

Where $\gamma$ is the coefficients vector and $\eta$ is the unobservable error term. Vector $\boldsymbol{\gamma}_{\boldsymbol{i}}$ contains the covariates that influence the outcome variable, and vector $\mathbf{w}_{\boldsymbol{i}}$ contains covariates that affect the treatment assignment. Based on our balance test, $\boldsymbol{\gamma}$ include age, gender, education, and civil status, while $\mathbf{w}_{\boldsymbol{i}}$ include all observable demographic and socioeconomic variables. The error terms are independent of $\boldsymbol{\gamma}_{\boldsymbol{i}}$ and $\mathbf{w}_{\boldsymbol{i}}$.

To obtain the ATE, we need to estimate the potential-outcome mean (POM) for treated $E({MH}_{1})$. The IPW estimator for the POM is $\frac{1}{N\Sigma_{i=1}^{N}}\cdot\frac{{MH}_{i}t_{i}}{p(x_{i}\boldsymbol{)}}$*,* where $p(x_{i})$ is the probability that $t_{i}=1$ as a function of the covariates $\boldsymbol{\gamma}_{\boldsymbol{i}}$*.* We consider a probit model for the treatment variable.

**Figure A1. Policy Change Timeline**


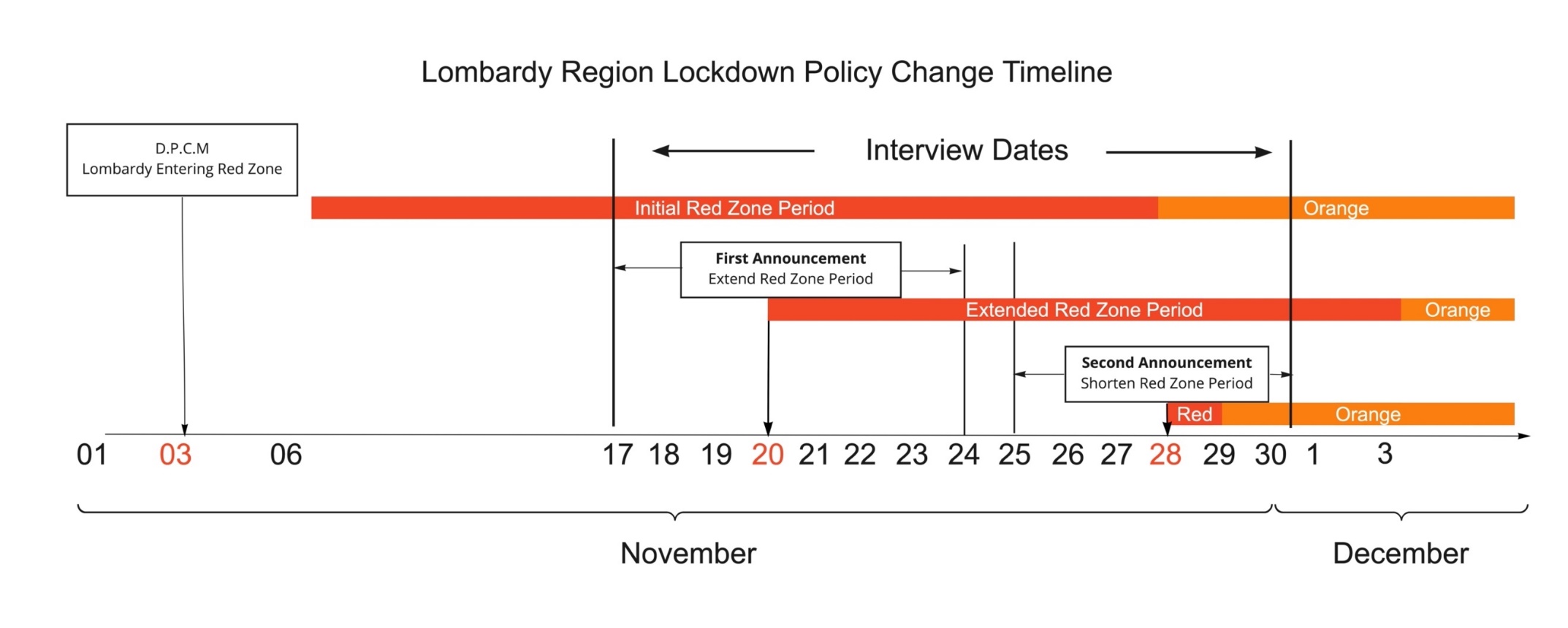


Note: D.P.C.M: Decreto del Presidente del Consiglio dei Ministri (Decree of the President of the Council of Ministers). This figure reports the timeline of the change in lockdown policies in the Lombardy region. The white boxes indicate the order of policy change; the red and orange rectangles represent the corresponding restrictive measure periods according to the respective policy/ordinance (red: maximum level of restriction; orange: intermediate level of restriction). The numbers that are in colour red indicate the dates of announcement. The interview period overlaps with two of the policy changes that occurred in November.

| **Table A1. Distribution of Number of Respondents by Interview Dates** | | | |
| --- | --- | --- | --- |
| Interview Date | Frequency | Percentage | Cumulative Percentage |
| 17-Nov-20 | 231 | 5.25 | 5.25 |
| 18-Nov-20 | 328 | 7.45 | 12.70 |
| 19-Nov-20 | 428 | 9.73 | 22.43 |
| 20-Nov-20 | 437 | 9.93 | 32.36 |
| 21-Nov-20 | 347 | 7.89 | 40.25 |
| 23-Nov-20 | 402 | 9.14 | 49.39 |
| 24-Nov-20 | 467 | 10.61 | 60.00 |
| 25-Nov-20 | 428 | 9.73 | 69.73 |
| 26-Nov-20 | 402 | 9.14 | 78.86 |
| 27-Nov-20 | 454 | 10.32 | 89.18 |
| 28-Nov-20 | 341 | 7.75 | 96.93 |
| 30-Nov-20 | 135 | 3.07 | 100.00 |

| **Table A2. First Announcement Balance Test, Immediate Effect** | | **Treatment Group** | | **Control Group** | | **Difference** |  |
| --- | --- | --- | --- | --- | --- | --- | --- |
|  |  | **N** | **%** | **N** | **%** | **chi-square** |  |
| **Outcome Variables** |  |  |  |  |  |  |  |
| Mental Health | Anxiety Symptoms | 147 | 33.6 | 600 | 27.2 | 7.37*** |  |
|  | Depressive Symptoms | 99 | 22.7 | 364 | 16.5 | 9.48*** |  |
|  | Poor-Quality Sleep | 64 | 14.7 | 315 | 14.3 | 0.04 |  |
| **Sociodemographic Variables** | |  |  |  |  |  |  |
| Age Category | 65-74 | 239 | 54.7 | 1,259 | 57.2 | 0.90 |  |
|  | 75+ | 198 | 45.3 | 944 | 42.9 |  |  |
| Gender | Male | 162 | 37.1 | 786 | 35.7 | 0.31 |  |
|  | Female | 275 | 62.9 | 1,417 | 64.3 |  |  |
| Education | No Title | 0 | 0.0 | 3 | 0.1 | 5.53 |  |
|  | Elementary Degree | 70 | 16.0 | 402 | 18.2 |  |  |
|  | Lower Secondary School Degree | 173 | 39.6 | 751 | 34.1 |  |  |
|  | Upper Secondary School Degree | 155 | 35.5 | 843 | 37.9 |  |  |
|  | University or Above | 39 | 8.9 | 213 | 9.7 |  |  |
| Civil Status | Married/cohabitant | 311 | 71.2 | 1,510 | 68.6 | 4.82 |  |
|  | Divorced/separated | 22 | 5.0 | 79 | 3.6 |  |  |
|  | Widower | 82 | 18.8 | 470 | 21.3 |  |  |
|  | Unmarried/Single | 22 | 5.0 | 144 | 6.5 |  |  |
| Income Level | Well above Italian Average | 1 | 0.2 | 5 | 0.2 | 2.49 |  |
|  | A little above Italian Average | 34 | 7.8 | 206 | 9.4 |  |  |
|  | Aligned with Italian Average | 318 | 72.8 | 1,625 | 73.8 |  |  |
|  | A little below Italian Average | 73 | 16.7 | 322 | 14.6 |  |  |
|  | Well below Italian Average | 11 | 2.5 | 45 | 2.0 |  |  |
| Covid | Had Covid | 17 | 3.9 | 100 | 4.5 | 0.36 |  |
|  | Never had Covid | 420 | 96.1 | 2,103 | 95.5 |  |  |
| Alone | Living in single household | 89 | 21.2 | 520 | 23.6 | 2.15 |  |
|  | Living with one or more people | 348 | 78.8 | 1,683 | 76.4 |  |  |
| Chronic Diseases | One or more chronic diseases | 362 | 82.8 | 1,794 | 81.4 | 0.48 |  |
|  | No | 75 | 17.2 | 409 | 18.6 |  |  |
| Number of Respondents |  | 437 |  | 2,203 |  |  |  |
| Notes: Treatment group include those interviewed on the announcement date November 20th, while the control group include those interviewed on November 18th, 19th, 21st, 22nd 23rd and 24th. We report the Pearson chi-square of the differences in all variables between treatment and control group; *** p<0.01, ** p<0.05, * p<0.1. | | | | | | |  |
|  |  |  |  |  |  |  |  |

| **Table A3. Second Announcement Balance Test, Immediate Effect** | | **Treatment Group** | | **Control Group** | | **Difference** |  |
| --- | --- | --- | --- | --- | --- | --- | --- |
|  |  | **N** | **%** | **N** | **%** | **chi-square** |  |
| **Outcome Variables** | |  |  |  |  |  |  |
| Mental Health | Anxiety Symptoms | 100 | 29.3 | 228 | 26.3 | 1.11 |  |
|  | Depressive Symptoms | 60 | 17.6 | 134 | 15.5 | 0.82 |  |
|  | Poor-Quality Sleep | 46 | 13.5 | 134 | 15.5 | 0.76 |  |
| **Sociodemographic Variables** | |  |  |  |  |  |  |
| Age Category | 75+ | 341 | 100.0 | 866 | 100.0 |  |  |
| Gender | Male | 166 | 48.7 | 295 | 34.1 | 22.14*** |  |
|  | Female | 175 | 51.3 | 571 | 65.9 |  |  |
| Education | No Title | 0 | 0.0 | 2 | 0.2 | 14.46*** |  |
|  | Elementary Degree | 57 | 16.7 | 230 | 26.6 |  |  |
|  | Lower Secondary School Degree | 128 | 37.5 | 296 | 34.2 |  |  |
|  | Upper Secondary School Degree | 118 | 34.6 | 262 | 30.3 |  |  |
|  | University or Above | 38 | 11.2 | 76 | 8.8 |  |  |
| Civil Status | Married/cohabitant | 234 | 68.6 | 586 | 67.7 | 8.91** |  |
|  | Divorced/separated | 7 | 2.1 | 14 | 1.6 |  |  |
|  | Widower | 73 | 21.4 | 229 | 26.4 |  |  |
|  | Unmarried/Single | 27 | 7.9 | 37 | 4.3 |  |  |
| Income Level | Well above Italian Average | 1 | 0.2 | 2 | 0.2 | 6.80 |  |
|  | A little above Italian Average | 27 | 7.8 | 54 | 6.2 |  |  |
|  | Aligned with Italian Average | 269 | 72.8 | 648 | 74.8 |  |  |
|  | A little below Italian Average | 42 | 16.7 | 150 | 17.3 |  |  |
|  | Well below Italian Average | 2 | 2.5 | 12 | 1.4 |  |  |
| Covid | Had Covid | 29 | 8.5 | 36 | 4.2 | 9.08*** |  |
|  | Never had Covid | 312 | 91.5 | 830 | 95.8 |  |  |
| Alone | Living in single household | 76 | 22.3 | 211 | 24.4 | 0.58 |  |
|  | Living with one or more people | 265 | 77.7 | 655 | 75.6 |  |  |
| Chronic Diseases | One or more chronic diseases | 287 | 84.2 | 742 | 85.7 | 0.45 |  |
|  | No | 54 | 15.8 | 124 | 14.3 |  |  |
| Number of Respondents | | 341 |  | 866 |  |  |  |
| Notes: Treatment group include those interviewed on the announcement date November 28th, while the control group include those interviewed on November 25th, 26st, 27th, and 30th. We report the Pearson chi-square of the differences in all variables between treatment and control group; *** p<0.01, ** p<0.05, * p<0.1. | | | | | | |  |
|  |  |  |  |  |  |  |  |

| **Table A4 First Announcement Balance Test, Rebound Effect** | | **Treatment Group** | | **Control Group** | | **Difference** |  |
| --- | --- | --- | --- | --- | --- | --- | --- |
|  |  | **N** | **%** | **N** | **%** | **chi-square** |  |
| **Outcome Variables** | |  |  |  |  |  |  |
| Mental Health | Anxiety Symptoms | 312 | 25.7 | 435 | 30.6 | 7.73*** |  |
|  | Depressive Symptoms | 186 | 15.3 | 277 | 19.5 | 7.83*** |  |
|  | Poor-Quality Sleep | 144 | 11.8 | 235 | 16.5 | 11.59*** |  |
| **Sociodemographic Variables** | |  |  |  |  |  |  |
| Age Category | 65-74 | 695 | 57.2 | 803 | 56.4 | 0.16 |  |
|  | 75+ | 521 | 42.9 | 621 | 43.6 |  |  |
| Gender | Male | 449 | 36.9 | 499 | 35.0 | 1.01 |  |
|  | Female | 767 | 63.1 | 925 | 65.0 |  |  |
| Education | No Title | 1 | 0.1 | 2 | 0.1 | 8.25* |  |
|  | Elementary Degree | 200 | 16.5 | 272 | 19.1 |  |  |
|  | Lower Secondary School Degree | 412 | 33.8 | 512 | 36.0 |  |  |
|  | Upper Secondary School Degree | 472 | 38.8 | 517 | 36.3 |  |  |
|  | University or Above | 131 | 10.8 | 121 | 8.5 |  |  |
| Civil Status | Married/cohabitant | 851 | 70.0 | 970 | 68.1 | 1.07 |  |
|  | Divorced/separated | 45 | 3.7 | 56 | 3.9 |  |  |
|  | Widower | 246 | 20.2 | 306 | 21.5 |  |  |
|  | Unmarried/Single | 74 | 6.1 | 92 | 6.5 |  |  |
| Income Level | Well above Italian Average | 4 | 0.3 | 2 | 0.1 | 6.60 |  |
|  | A little above Italian Average | 117 | 9.6 | 123 | 0.6 |  |  |
|  | Aligned with Italian Average | 868 | 71.4 | 1,075 | 75.5 |  |  |
|  | A little below Italian Average | 198 | 16.3 | 197 | 13.8 |  |  |
|  | Well below Italian Average | 29 | 2.4 | 27 | 1.9 |  |  |
| Covid | Had Covid | 50 | 4.1 | 67 | 4.7 | 0.55 |  |
|  | Never had Covid | 1,166 | 95.9 | 1,358 | 95.3 |  |  |
| Alone | Living in single household | 277 | 22.8 | 332 | 23.3 | 0.11 |  |
|  | Living with one or more people | 939 | 77.2 | 1,092 | 76.7 |  |  |
| Chronic Diseases | One or more chronic diseases | 997 | 82.0 | 1,159 | 81.4 | 0.16 |  |
|  | No | 219 | 18.0 | 265 | 18.6 |  |  |
| Number of Respondents | | 1,216 |  | 1,424 |  |  |  |
| Note: Treatment group include those interviewed after the first announcement date, November 21st to 24th, while the control group include those interviewed before and on the first announcement date, November 17th to 20th. We report the Pearson chi-square of the differences in all variables between treatment and control group; *** p<0.01, ** p<0.05, * p<0.1. | | | | | | |  |
|  |  |  |  |  |  |  |  |

| **Table A5. Covariate Coefficients from Table 1, First Announcement, Model Adjusted for Confounding Factors** | | | | |
| --- | --- | --- | --- | --- |
|  |  | (1) | (2) | (3) |
| VARIABLES |  | Anxiety | Depression | Poor-quality sleep |
| Over 75 Years Old | | 0.011 | 0.022 | -0.002 |
|  |  | (-0.023 - 0.045) | (-0.009 - 0.054) | (-0.039 - 0.035) |
| Female |  | 0.115*** | 0.057*** | 0.042*** |
|  |  | (0.083 - 0.147) | (0.030 - 0.085) | (0.019 - 0.065) |
| Province FE |  | -0.000 | -0.000 | 0.000 |
|  |  | (-0.001 - 0.001) | (-0.001 - 0.000) | (-0.000 - 0.000) |
| Education | No Title | Reference | | |
|  | Elementary Degree | 0.136** | 0.056* | 0.102*** |
|  |  | (0.004 - 0.268) | (-0.009 - 0.122) | (0.061 - 0.142) |
|  | Lower Secondary School Degree | 0.197** | 0.114*** | 0.093*** |
|  |  | (0.049 - 0.346) | (0.048 - 0.180) | (0.063 - 0.122) |
|  | Upper Secondary School Degree | 0.187** | 0.095*** | 0.136*** |
|  |  | (0.050 - 0.325) | (0.046 - 0.144) | (0.103 - 0.168) |
|  | University or Above | 0.212*** | 0.123*** | 0.115*** |
|  |  | (0.096 - 0.327) | (0.061 - 0.185) | (0.055 - 0.175) |
| Civil Status | Married | Reference | | |
|  | Divorced/separated | 0.101** | 0.037 | 0.013 |
|  |  | (0.000 - 0.201) | (-0.031 - 0.105) | (-0.060 - 0.085) |
|  | Widower | -0.052 | -0.003 | 0.043* |
|  |  | (-0.164 - 0.060) | (-0.041 - 0.034) | (-0.009 - 0.094) |
|  | Unmarried/Single | 0.007 | -0.002 | 0.073** |
|  |  | (-0.062 - 0.076) | (-0.067 - 0.063) | (0.001 - 0.145) |
| Income Level | Well above Italian Average | Reference | | |
|  | A little above Italian Average | 0.043 | -0.055 | -0.059 |
|  |  | (-0.291 - 0.376) | (-0.390 - 0.281) | (-0.327 - 0.210) |
|  | Aligned with Italian Average | 0.061 | -0.037 | -0.057 |
|  |  | (-0.263 - 0.385) | (-0.368 - 0.293) | (-0.327 - 0.213) |
|  | A little below Italian Average | 0.274 | 0.142 | -0.069 |
|  |  | (-0.076 - 0.623) | (-0.208 - 0.491) | (-0.340 - 0.203) |
|  | Well below Italian Average | 0.625*** | 0.367* | -0.042 |
|  |  | (0.284 - 0.966) | (-0.008 - 0.741) | (-0.339 - 0.254) |
| Covid |  | 0.023 | 0.049 | 0.041 |
|  |  | (-0.055 - 0.101) | (-0.034 - 0.132) | (-0.023 - 0.105) |
| Alone |  | 0.005 | 0.008 | 0.014 |
|  |  | (-0.114 - 0.125) | (-0.034 - 0.050) | (-0.054 - 0.082) |
| Chronic Diseases | | 0.081*** | 0.078*** | 0.063*** |
|  |  | (0.053 - 0.109) | (0.045 - 0.111) | (0.035 - 0.091) |
| Notes: Robust standard errors clustered at provincial level; Standard errors in parentheses; *** p<0.01, ** p<0.05, * p<0.1 | | | | |

| **Table A6. Covariate Coefficients from Table 1, Second Announcement, Model Adjusted for Confounding Factors** | | | | |
| --- | --- | --- | --- | --- |
|  |  | (1) | (2) | (3) |
| VARIABLES |  | Anxiety | Depression | Poor Quality Sleep |
| Female |  | 0.054 | 0.006 | 0.028 |
|  |  | (-0.014 - 0.123) | (-0.050 - 0.062) | (-0.021 - 0.078) |
| Province FE |  | 0.001** | -0.000 | 0.001** |
|  |  | (0.000 - 0.001) | (-0.000 - 0.000) | (0.000 - 0.001) |
| Education | No Title | Reference | | |
|  | Elementary Degree | 0.290*** | 0.251*** | 0.184*** |
|  |  | (0.221 - 0.360) | (0.190 - 0.313) | (0.096 - 0.272) |
|  | Lower Secondary School Degree | 0.262*** | 0.168*** | 0.169*** |
|  |  | (0.195 - 0.330) | (0.080 - 0.256) | (0.083 - 0.256) |
|  | Upper Secondary School Degree | 0.176*** | 0.104** | 0.166*** |
|  |  | (0.118 - 0.234) | (0.021 - 0.187) | (0.079 - 0.254) |
|  | University or Above | 0.256*** | 0.067 | 0.217** |
|  |  | (0.179 - 0.332) | (-0.058 - 0.193) | (0.054 - 0.381) |
| Civil Status | Married | Reference | | |
|  | Divorced/separated | -0.008 | 0.026 | 0.020 |
|  |  | (-0.335 - 0.318) | (-0.152 - 0.203) | (-0.129 - 0.169) |
|  | Widower | -0.008 | 0.025 | 0.000 |
|  |  | (-0.183 - 0.168) | (-0.103 - 0.152) | (-0.105 - 0.105) |
|  | Unmarried/Single | -0.012 | 0.073 | -0.004 |
|  |  | (-0.237 - 0.212) | (-0.072 - 0.219) | (-0.112 - 0.104) |
| Income Level | Well above Italian Average | Reference | | |
|  | A little above Italian Average | -0.145 | 0.204*** | 0.111* |
|  |  | (-0.808 - 0.518) | (0.088 - 0.319) | (-0.007 - 0.229) |
|  | Aligned with Italian Average | -0.152 | 0.093** | 0.074** |
|  |  | (-0.782 - 0.478) | (0.008 - 0.178) | (0.002 - 0.146) |
|  | A little below Italian Average | -0.046 | 0.131*** | 0.178** |
|  |  | (-0.695 - 0.602) | (0.058 - 0.205) | (0.049 - 0.308) |
|  | Well below Italian Average | 0.189 | 0.244 | 0.206 |
|  |  | (-0.336 - 0.713) | (-0.115 - 0.603) | (-0.075 - 0.486) |
| Covid |  | -0.070 | 0.063* | 0.095 |
|  |  | (-0.168 - 0.029) | (-0.011 - 0.137) | (-0.058 - 0.247) |
| Alone |  | -0.029 | -0.019 | 0.014 |
|  |  | (-0.188 - 0.130) | (-0.117 - 0.079) | (-0.067 - 0.094) |
| Chronic Diseases | | 0.116*** | 0.066*** | 0.081*** |
|  |  | (0.042 - 0.190) | (0.033 - 0.100) | (0.033 - 0.129) |
| Notes: Robust standard errors clustered at provincial level; Standard errors in parentheses; *** p<0.01, ** p<0.05, * p<0.1 | | | | |

| **Table A7. Covariate Coefficients from Table 2, First Announcement, Model Adjusted for Confounding Factors** | | | | |
| --- | --- | --- | --- | --- |
|  |  | (1) | (2) | (3) |
| VARIABLES |  | Anxiety | Depression | Poor-quality sleep |
| Over 75 Years Old | | 0.012 | 0.023 | -0.002 |
|  |  | (-0.022 - 0.046) | (-0.007 - 0.054) | (-0.040 - 0.035) |
| Female |  | 0.114*** | 0.056*** | 0.042*** |
|  |  | (0.081 - 0.146) | (0.030 - 0.083) | (0.021 - 0.063) |
| Province FE |  | -0.000 | -0.000 | 0.000 |
|  |  | (-0.001 - 0.001) | (-0.001 - 0.000) | (-0.000 - 0.000) |
| Education | No Title | Reference | | |
|  | Elementary Degree | 0.148* | 0.067 | 0.106*** |
|  |  | (-0.022 - 0.319) | (-0.033 - 0.168) | (0.057 - 0.156) |
|  | Lower Secondary School Degree | 0.212** | 0.128** | 0.099*** |
|  |  | (0.021 - 0.403) | (0.028 - 0.228) | (0.041 - 0.156) |
|  | Upper Secondary School Degree | 0.203** | 0.110** | 0.143*** |
|  |  | (0.027 - 0.380) | (0.029 - 0.191) | (0.090 - 0.197) |
|  | University or Above | 0.230*** | 0.139*** | 0.125*** |
|  |  | (0.077 - 0.382) | (0.043 - 0.236) | (0.064 - 0.186) |
| Civil Status | Married | Reference | | |
|  | Divorced/separated | 0.101* | 0.038 | 0.010 |
|  |  | (-0.001 - 0.203) | (-0.036 - 0.111) | (-0.065 - 0.086) |
|  | Widower | -0.055 | -0.006 | 0.040 |
|  |  | (-0.167 - 0.058) | (-0.042 - 0.031) | (-0.011 - 0.092) |
|  | Unmarried/Single | 0.003 | -0.006 | 0.070* |
|  |  | (-0.064 - 0.071) | (-0.071 - 0.060) | (-0.001 - 0.141) |
| Income Level | Well above Italian Average | Reference | | |
|  | A little above Italian Average | 0.032 | -0.064 | -0.067 |
|  |  | (-0.305 - 0.369) | (-0.405 - 0.278) | (-0.352 - 0.219) |
|  | Aligned with Italian Average | 0.050 | -0.046 | -0.066 |
|  |  | (-0.275 - 0.376) | (-0.383 - 0.290) | (-0.351 - 0.220) |
|  | A little below Italian Average | 0.268 | 0.137 | -0.074 |
|  |  | (-0.086 - 0.622) | (-0.218 - 0.492) | (-0.360 - 0.213) |
|  | Well below Italian Average | 0.620*** | 0.363* | -0.047 |
|  |  | (0.273 - 0.967) | (-0.021 - 0.747) | (-0.358 - 0.264) |
| Covid |  | 0.020 | 0.047 | 0.039 |
|  |  | (-0.056 - 0.096) | (-0.038 - 0.131) | (-0.025 - 0.104) |
| Alone |  | 0.006 | 0.008 | 0.016 |
|  |  | (-0.114 - 0.126) | (-0.032 - 0.049) | (-0.053 - 0.084) |
| Chronic Diseases | | 0.082*** | 0.079*** | 0.064*** |
|  |  | (0.053 - 0.111) | (0.045 - 0.113) | (0.034 - 0.093) |
| Notes: Robust standard errors clustered at provincial level; Standard errors in parentheses; *** p<0.01, ** p<0.05, * p<0.1 | | | | |

| **Table A8. Robustness Check, Immediate Effect including Interview-day Fixed Effects** | | | | |
| --- | --- | --- | --- | --- |
|  |  | (1) | (2) | (3) |
| Models |  | Anxiety Symptoms | Depressive Symptoms | Poor Quality Sleep |
| First Announcement | Adjusted for Confounding Factors | 0.002 | 0.002 | -0.034 |
|  |  | (-0.054 - 0.059) | (-0.046 - 0.049) | (-0.086 - 0.017) |
|  | Inverse Probability Weighting | 0.052** | 0.048*** | 0.003 |
|  |  | (0.008 - 0.096) | (0.025 - 0.072) | (-0.041 - 0.047) |
|  | Number of Respondents | 2,640 | 2,640 | 2,640 |
|  |  |  |  |  |
| Second Announcement | Adjusted for Confounding Factors | 0.046 | 0.061** | -0.007 |
|  |  | (-0.051 - 0.144) | (0.007 - 0.115) | (-0.057 - 0.043) |
|  | Inverse Probability Weighting | 0.057** | 0.033*** | -0.019 |
|  |  | (0.010 - 0.105) | (0.013 - 0.053) | (-0.051 - 0.013) |
|  | Number of Respondents | 1,207 | 1,207 | 1,207 |
| Robust standard errors clustered at provincial level; Standard errors in parentheses; *** p<0.01, ** p<0.05, * p<0.1 | | | | |
| Outcome Measurement Units: having anxiety symptom, having depressive symptom, having poor quality sleep | | | | |

| **Table A9. Probit Analysis with Risk Adjustment, Interview Days and Selected Variables** | | | | |
| --- | --- | --- | --- | --- |
|  |  | (1) | (2) | (3) |
| Marginal Effects at Mean |  | Anxiety Symptoms | Depressive Symptoms | Poor-quality sleep |
| Interview Days | 17 | 0.330*** | 0.400*** | 0.435*** |
|  |  | (0.120) | (0.0958) | (0.122) |
|  | 18 | 0.228*** | 0.168 | 0.256** |
|  |  | (0.0784) | (0.107) | (0.104) |
|  | 19 | 0.242*** | 0.270*** | 0.440*** |
|  |  | (0.0767) | (0.0954) | (0.104) |
|  | **20** | **0.335***** | **0.389***** | **0.281**** |
|  |  | **(0.0773)** | **(0.0606)** | **(0.115)** |
|  | 21 | 0.185*** | 0.261*** | 0.127 |
|  |  | (0.0699) | (0.0869) | (0.0796) |
|  | 23 | Reference | | |
|  | 24 | 0.129* | 0.0860 | 0.240** |
|  |  | (0.0762) | (0.0675) | (0.119) |
|  | 25 | 0.162 | 0.0427 | 0.247*** |
|  |  | (0.127) | (0.185) | (0.0923) |
|  | 26 | 0.155 | 0.270** | 0.317*** |
|  |  | (0.124) | (0.115) | (0.0910) |
|  | 27 | 0.158** | 0.0723 | 0.219* |
|  |  | (0.0697) | (0.109) | (0.120) |
|  | **28** | **0.315***** | **0.269**** | **0.217**** |
|  |  | **(0.0626)** | **(0.131)** | **(0.0925)** |
|  | 30 | 0.133 | 0.276* | 0.227** |
|  |  | (0.169) | (0.167) | (0.115) |
| Income Level | Well above Italian Average | Reference | | |
|  | A little above Italian Average | 0.125 | 0.238 | 0.130 |
|  |  | (0.620) | (0.486) | (0.481) |
|  | Aligned with Italian Average | 0.188 | 0.208 | 0.144 |
|  |  | (0.619) | (0.489) | (0.477) |
|  | A little below Italian Average | 0.715 | 0.682 | 0.266 |
|  |  | (0.649) | (0.502) | (0.490) |
|  | Well below Italian Average | 1.652*** | 1.312*** | 0.343 |
|  |  | (0.608) | (0.509) | (0.412) |
| Covid | Yes | 0.0343 | 0.195** | 0.331*** |
|  |  | (0.0988) | (0.0846) | (0.0579) |
| Chronic Disease | Yes | 0.296*** | 0.393*** | 0.289*** |
|  |  | (0.0394) | (0.0350) | (0.0406) |
| Number of Respondents |  | 4,400 | 4,400 | 4,400 |
| Notes: Robust standard errors clustered at provincial level; Standard errors in parentheses; *** p<0.01, ** p<0.05, * p<0.1 | | | | |
| Outcome Measurement Units: having anxiety symptom, having depressive symptom, having poor-quality sleep | | | | |

| **Table A10. (continued) Covariate Coefficients, Probit Model with Risk-Adjustment** | | | | |  | |
| --- | --- | --- | --- | --- | --- | --- |
|  | | (1) | (2) | (3) | |  |
| Marginal Effects at Mean | | Anxiety Symptoms | Depressive Symptoms | Poor-quality sleep | |  |
| Over 85 Years Old | | -0.0164 | 0.00746 | 0.0176 | |  |
|  |  | (0.0355) | (0.0499) | (0.0671) | |  |
| Female |  | 0.279*** | 0.156*** | 0.167*** | |  |
|  |  | (0.0392) | (0.0427) | (0.0405) | |  |
| Province FE |  | 0.000490 | -0.000251 | 0.00102 | |  |
|  |  | (0.000757) | (0.000623) | (0.000658) | |  |
| Education | No Title |  |  |  |  |  |
|  | Elementary Degree | -0.160* | 0.0165 | -0.102 | |  |
|  |  | (0.0868) | (0.0786) | (0.0915) | |  |
|  | Lower Secondary School Degree | -0.0727 | 0.0436 | -0.142 | |  |
|  |  | (0.0618) | (0.0565) | (0.103) | |  |
|  | Upper Secondary School Degree | -0.178*** | -0.0772 | -0.0244 | |  |
|  |  | (0.0548) | (0.0906) | (0.0988) | |  |
| Civil Status | Married |  |  |  |  |  |
|  | Divorced/separated | 0.247** | 0.0971 | 0.118 | |  |
|  |  | (0.123) | (0.117) | (0.110) | |  |
|  | Widower | -0.125 | -0.0464 | 0.121 | |  |
|  |  | (0.140) | (0.101) | (0.0996) | |  |
|  | Unmarried/Single | -0.0594 | 0.000166 | 0.141* | |  |
|  |  | (0.0977) | (0.166) | (0.0836) | |  |
| Living Alone |  | 0.00342 | 0.0964 | 0.0405 | |  |
|  |  | (0.140) | (0.104) | (0.0608) | |  |
| Constant |  | -1.383** | -1.937*** | -1.869*** | |  |
|  |  | (0.563) | (0.431) | (0.552) | |  |

Notes: Robust standard errors clustered at provincial level; Standard errors in parentheses; *** p<0.01, ** p<0.05, * p<0.1

1. Presidenza del Consiglio dei Ministri (https://www.gazzettaufficiale.it/eli/id/2020/11/04/20A06109/sg) [↑](#footnote-ref-1)
2. Ordinanza Ministero di Salute – Conferenza Stampa (<https://www.salute.gov.it/portale/news/p3_2_4_1_1.jsp?lingua=italiano&menu=salastampa&p=comunicatistampa&id=5678>) [↑](#footnote-ref-2)
3. Nuova Ordinanza Ministero di Salute (<https://www.salute.gov.it/portale/nuovocoronavirus/dettaglioNotizieNuovoCoronavirus.jsp?lingua=italiano&id=5197>) [↑](#footnote-ref-3)
